# Directional Moisture Transport in Compositionally Graded Multilayer Membranes

**DOI:** 10.1021/acsapm.5c03299

**Published:** 2025-12-08

**Authors:** Natthakan Rattanaphong, Luca Grillo, Christoph Weder, Stephan Thierry Dubas

**Affiliations:** † The Petroleum and Petrochemical College, 26683 Chulalongkorn University, Bangkok 10330, Thailand; ‡ Adolphe Merkle Institute University of Fribourg, Chemin des Verdier 4, 1700 Fribourg, Switzerland; § Center of Excellence on Petrochemical and Materials Technology, Bangkok 10330, Thailand; ∥ Machine Learning for Polymers and Materials Discovery Research Unit, The Petroleum and Petrochemical College, Chulalongkorn University, Bangkok 10330, Thailand

**Keywords:** directional moisture transport, asymmetric membrane, dense membrane, water permeability, poly(styrene-*b*-butadiene-*b*-styrene), polymer blend

## Abstract

Directional water or moisture transport, which is vital for many living species, can be achieved by several operating principles and material designs, including dense membranes with a polarity gradient in the transverse direction. Here, we present a straightforward approach to create artificial membranes that mimic this design and offer highly directional moisture transport. The reported membranes consist of a hydrophobic poly­(styrene-*b*-butadiene-*b*-styrene) (SBS) layer and a hydrophilic layer made from a terpolymer (TP) of 2-hydroxyethyl methacrylate (HEMA), 2-hydroxyethyl acrylate (HEA), and 2-ethylhexyl methacrylate (EHMA) or blends of this terpolymer and SBS. When the hydrophilic TP-rich side of the membranes is exposed to moisture, the TP or TP blend swells and becomes plasticized, resulting in increased water permeability. Such plasticization is limited or absent when the SBS side is exposed to moisture. The mechanical robustness and asymmetric moisture-transport characteristics of such membranes were improved by employing an SBS-TP blend as an interfacial adhesive layer between the SBS and TP layers. Such trilayer membranes do not exhibit any signs of delamination even after extended exposure to moisture. They display highly directional transport with an asymmetry factor of 4.0 ± 1.1, outperforming many of the previously investigated graded membranes.

## Graphical abstract

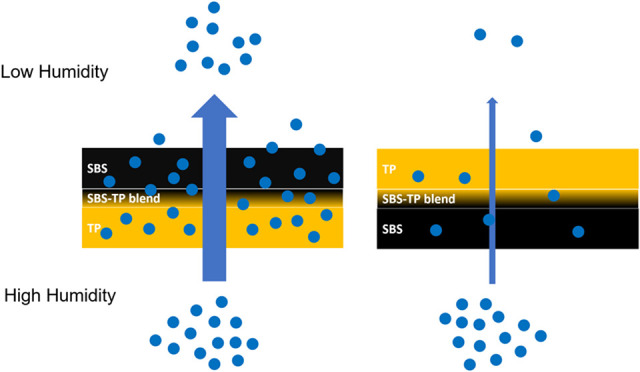

## Introduction

The directional transport of water or moisture plays a crucial role in several living organisms as a mechanism for collecting and retaining water.^1,2^ This phenomenon also finds industrial applications, for example, in the context of moisture management,^3^ water harvesting,^4^ functional textiles,^5–8^ packaging,^9^ and separation processes. Membranes that allow directional water transport can be broadly classified into porous and dense, each utilizing distinct mechanisms.^10,11^ Porous membranes are mainly used for the directional transport of liquid water. The direction of transport is controlled by regulating pore size, density, and a wettability gradient,^12^ which governs the flow rate and selectivity, resulting in the unidirectional transport of permeants.^13^ In contrast, moisture permeation through dense membranes is governed by the sorption–diffusion mechanism, in which water vapor molecules first sorb onto the membrane surface, diffuse through the polymer matrix, and finally desorb on the opposite side.^14–16^ Directional moisture transport occurs when the magnitude of the permeation rate of water vapor through the same membrane under the same external conditions (humidity gradient, temperature, etc.) depends on the side from which the moisture penetrates the membrane.^15,16^

To achieve such directionality, dense membranes must have an asymmetric structure, specifically a transversal polarity gradient, and they must contain at least one component with a water-vapor-pressure-dependent permeability coefficient.^15–19^ When the membrane side rich in the latter is exposed to moisture, the permeability of this membrane portion is generally higher than that if the opposite side is exposed to the permeant. Such spatially heterogeneous structures can be accessed by fabricating multilayer laminates or producing films that exhibit a compositional gradient. While laminated structures are easy to fabricate and have been widely used to achieve asymmetric mass transport,^15,20,21^ delamination is a common problem. Membranes that exhibit a gradual variation in composition, structure, or functionality along their transversal direction can overcome this problem, but their fabrication is generally more complicated.^22,23^ Processes that have been used to produce graded membranes include sedimental casting,^24^ controlled cross-linking,^25^ and gradient polymerization.^21,26^ Due to the seamless gradient and the absence of “sharp” interfaces, the mechanical coherence of such membranes is generally exquisite.

Previous studies have demonstrated the potential of gradient structures to induce asymmetric water transport. Kamtsikakis et al.^24^ successfully fabricated compositionally graded membranes using a solvent-casting/sedimentation approach, which generated a spatial gradient in the concentration of cellulose nanocrystals (CNC) dispersed in a hydrophobic matrix made of poly­(styrene-*b*-butadiene-*b*-styrene) (SBS). The study showed that the water permeability was higher in the direction from the CNC-rich side to the hydrophobic SBS side, indicating an asymmetric transport behavior. The asymmetry factor (AF, defined as the ratio of the water permeabilities measured in the two directions) increased with the CNC content and membrane thickness, reaching a maximum value of 2.5. Building on this nanocomposite approach, Grillo and Weder^27^ developed asymmetric nanocomposite membranes that combine electro-spun poly­(vinyl alcohol) (PVA) nanofibers and SBS. Replacing the CNCs with PVA nanofibers rendered the water transport not only asymmetric with an AF of up to 2.3 but also switchable. When the PVA-rich side of the membranes is exposed to high relative humidity (RH), the PVA is plasticized and the permeability of the membrane increases. Such plasticization is limited when the SBS-rich side is exposed to humid conditions. In a recent follow-up study, Grillo and Weder investigated bilayer structures comprising a thick PVA layer and a thin SBS layer, reporting that such layered membranes exhibit much higher asymmetry factors (up to 5.8) than the compositionally graded membranes made with the same components.^28^ This finding is supported by theoretical work, which suggests that the optimal membrane configuration to maximize the AF is a binary laminate.^29^

Due to the semicrystalline nature of PVA and the limited permeability caused by the crystalline domains,^30,31^ we set out to replace PVA with a fully amorphous hydrophilic polymer. For this purpose, a terpolymer formed by the free-radical polymerization of 2-hydroxyethyl methacrylate (HEMA), 2-hydroxyethyl acrylate (HEA), and 2-ethylhexyl methacrylate (EHMA) (poly­(HEMA-*co*-HEA-*co*-EHMA), hereafter referred to as “TP”), which was originally developed by Monney et al. for mechanically adaptive implants,^32^ was selected. This polymer is glassy and rigid when dry but softens upon exposure to moisture, which causes a significant reduction in the glass transition temperature (*T*_g_), making it a suitable candidate for the targeted membranes. Notably, TP exhibits a *T*_g_ of 92 ± 2.5 °C in the dry state, which decreases to 4 ± 1 °C upon immersion in water, with only modest swelling. Prior studies showed that SBS blends with glassy hydrophilic polymers, such as poly­(lactic acid) (PLA), phase-separate, as expected. The morphologies observed range from sea–island to cocontinuous structures, depending on the composition, and this strongly influences the mechanical properties and other characteristics. For example, SBS-PLA blends exhibit a higher stiffness and lower extensibility as the PLA content increases, while shape memory effects are optimized at intermediate compositions.^33^ Based on these findings, we surmised that blending SBS and TP could afford suitable materials for membranes with moisture-dependent water permeability (WP), in which the SBS domains provide mechanical resilience, and the TP forms switchable moisture-transport channels.

## Experimental Section

### Materials

Poly­(styrene-*b*-butadiene-*b*-styrene) (SBS, 30 wt % styrene, weight-average molecular weight, *M*_w_, = 140 000 g/mol) was purchased from Sigma-Aldrich. Tetrahydrofuran (THF, analytical reagent grade, 99.5%) was purchased from LOBA CHEMIE PVT Ltd. 2-Hydroxyethyl methacrylate (HEMA), 2-hydroxyethyl acrylate (HEA), 2-ethylhexyl methacrylate (EHMA), hydroquinone, and 2,2′-azobis­(isobutyronitrile) (AIBN) were procured from Sigma-Aldrich. Dodecanethiol, ethanol (EtOH), dioxane, diethyl ether, and hexane (all solvent reagent grade) were purchased from Thermo Fisher.

### Synthesis of Poly­(HEMA-*co*-HEA-*co*-EHMA) (TP)

The synthesis of poly­(HEMA-*co*-HEA-*co*-EHMA) (TP) was conducted following the protocol reported previously^32^ (Figure S1). 2-Hydroxyethyl methacrylate (33 mL, 60 mol %), 2-hydroxyethyl acrylate (13 mL, 25 mol %), 2-ethylhexyl methacrylate (15 mL, 15 mol %), dodecanethiol (109 μL, 0.1 mol %), and ethanol (220 mL) were combined in a 500 mL two-necked round-bottom flask with a magnetic stir bar, a septum, and a reflux condenser, and the mixture was purged for 90 min with N_2_. The mixture was heated to 60 °C and stirred for 30 min before a solution of AIBN (1.49 g) in dioxane (15 mL) was added. The homogeneous reaction mixture was then stirred at 60 °C under a constant nitrogen flow for 3 h, before ca. 0.5 g of hydroquinone was added to terminate the reaction. The resulting polymer was precipitated twice into a 9:1 v/v mixture of diethyl ether and hexane, collected by filtration, and dried overnight in a vacuum oven at 60 °C. Between the two precipitations, the reaction product was dissolved in ethanol (150 mL). TP was obtained as a white solid (33.4 g, 54%). The analysis of the ^1^H NMR spectrum (Figure S2) according to the reported procedure^32^ resulted in HEMA, HEA, and EHMA fractions in the copolymer of 55, 28, and 17 mol %, respectively. Size exclusion chromatography (Figure S3) affords a number-average molecular weight (*M*_n_) of 56,000 g/mol and a dispersity (Đ) of 1.9, while differential scanning calorimetry (Figure S4) reveals a glass transition temperature (*T*_g_) of 62 °C.

### Fabrication of Neat SBS Films

A solution of SBS (0.36 g) in THF (25 mL) was cast into a round poly­(tetrafluoroethylene) (PTFE) mold with a diameter of 8 cm, and the solvent was allowed to evaporate overnight at room temperature. The SBS films thus obtained were further dried in a vacuum oven at 60 °C for 24 h. To ensure uniform thickness, the resulting films were placed between two PTFE sheets and compression-molded in a hot press at 130 °C with a load of 4 tons for 7 min. Spacers of 150 μm were applied to produce films with a thickness of 158 ± 9 μm.

### Fabrication of SBS-TP_*x*_ Blend Films

TP and SBS (see Table 1 for compositions) were dissolved in THF (25 mL) under stirring; the solutions were cast into round PTFE molds with a diameter of 8 cm, and the solvent was allowed to evaporate overnight at room temperature. The SBS-TP blend films thus obtained were further dried in a vacuum oven at 60 °C for 24 h. To ensure uniform thickness, the resulting films were placed between two PTFE sheets and compression-molded in a hot press at 130 °C with a load of 4 tons for 7 min. Spacers with a thickness of 150 μm were applied to produce films, with the thickness reported in Table 1. The composition of these blend films is indicated by SBS-TP_*x*_, where the subscript *x* denotes the weight fraction of TP.

**Table 1:** Composition and Thickness of the Various SBS-TP_*x*_ Blend Films Made

sample name	weight of SBS (g)	weight of TP (g)	TP fraction (wt %)	total mass (g)	thickness (μm)
neat SBS	0.36	0.00	0	0.36	158 ± 9
SBS-TP_10_	0.36	0.04	10	0.40	137 ± 17
SBS-TP_23_	0.36	0.11	23	0.47	148 ± 14
SBS-TP_33_	0.36	0.18	33	0.54	154 ± 9
SBS-TP_41_	0.36	0.25	41	0.61	170 ± 15
neat TP	0	0.52	100	0.52	146 ± 12

### Fabrication of Multilayer Membranes

To fabricate the SBS/TP or SBS/SBS-TP_*x*_ bilayer membrane, the neat SBS and the TP or SBS-TP_*x*_ blend films prepared as described above were placed on top of each other and subjected to compression molding under the same conditions as those applied for the fabrication of the single-layer films, but spacers with a thickness of 300 μm were applied to control the thickness of the bilayer membranes (Table S1). An SBS/SBS-TP_23_/TP triple-layer membrane was prepared in a similar manner. In this case, a 50 μm thick SBS-TP_23_ film was produced by compression molding the blend using a 50 μm thick copper sheet as a spacer. This SBS-TP_23_ film was then sandwiched between a ca. 150 μm thick SBS and a ca. 150 μm thick TP film, and a 300 ± 4 μm thick trilayer membrane was fabricated by compression molding, also using spacers with a thickness of 300 μm.

#### Thickness Measurements

The thickness of all films and membranes was measured using a digital micrometer (INSIZE 3109–25A). Ten thickness measurements were taken at random spots across the membrane, and the reported values are the average ± standard deviation.

#### Scanning Electron Microscopy (SEM)

The morphology of the SBS, TP, and SBS-TP blend single-layer films and of the multilayer membranes was investigated by SEM using a Hitachi S-4800 scanning electron microscope. Before testing, the membranes were dried in an oven at 60 °C for 3 d. Samples were prepared via cryofracturing in liquid nitrogen and mounted on stubs using carbon tape. The samples were subsequently coated with a thin layer of platinum. The SEM images were obtained using an acceleration voltage of 5 kV.

#### Optical Microscopy

Optical microscopy of the films and membranes was carried out using a customized microscope (ZEISS Axio Scope.A1) fitted with a CCD camera (Point Gray GS3-U3–28S5C–C) calibrated against a standard white diffuser and an LED white lamp as the light source. Micrographs were captured in reflection mode in a bright field configuration using a 50X objective (Zeiss LD EC Epiplan-Neofluar, NA = 0.8).

#### Dynamic Mechanical Analysis (DMA)

DMA experiments were conducted on a TA Instruments Q800 DMA in tensile mode over a temperature range of −100 to 150 °C. Samples were equilibrated for 5 min at −100 °C, and a heating rate of 5 °C/min, an amplitude of 15 μm, and a frequency of 1 Hz were applied. Rectangular specimens with a length of ca. 15 mm, a width of ca. 5 mm, and a thickness of ca. 150 μm were cut from the films and membranes and kept in a desiccator under vacuum prior to testing. All DMA data reported are averages from triplicate measurements with standard deviations, and representative experiments are presented.

#### Tensile Testing

Tensile tests were conducted by utilizing a Zwick/Roell Z010 tensile tester equipped with a 200 N load cell. Samples in the shape of dog bones were prepared from the films and membranes by using a die cutter (Zwick/Roell) and kept in a desiccator under vacuum prior to testing. To assess the influence of moisture on the mechanical resistance of the trilayer membranes, samples were conditioned for 1 week in an incubator maintained at 98% relative humidity using a saturated K_2_SO_4_ solution.^34^ The stress–strain measurements were performed under ambient conditions (23 °C, relative humidity of ca. 50%) at a strain rate of 50 mm/min.

#### Size Exclusion Chromatography (SEC)

Size exclusion chromatography (SEC) experiments were performed on an Agilent 1200 series HPLC system equipped with an Agilent PLgel mixed guard column (particle size = 5 μm) and two Agilent PLgel mixed-D columns (ID = 7.5 mm, L = 300 mm, and particle size = 5 μm). Signals were recorded by using a UV detector (Agilent 1200 series), an Optilab REX interferometric refractometer, and a miniDawn TREOS light scattering detector (Wyatt Technology Corp.). Samples were run by using THF as the eluent at 30 °C and a flow rate of 1.0 mL/min. Data analyses were carried out on Astra software (Wyatt Technology Corp.), and molecular weights were determined based on narrow molecular weight poly­(methyl methacrylate) standards calibration.

#### Nuclear Magnetic Resonance (NMR) Spectroscopy

^1^H NMR spectroscopy was carried out at 297.2 K on a Bruker Avance DPX 400 spectrometer at a frequency of 400.19 MHz. Spectra were calibrated to the residual solvent peak of DMSO-d6 (2.50 ppm). Data were evaluated with the MestReNova software suite (v 12.0), and all chemical shifts δ are reported in parts per million (ppm) with coupling constant in Hz (multiplicity: s = singlet, d = doublet, dd = double doublet, t = triplet, m = multiplet, br = broad signal).

#### Differential Scanning Calorimetry (DSC)

DSC was conducted on a Mettler Toledo DSC 2 STAR system under a nitrogen atmosphere from −25 to 180 °C with heating and cooling rates of 10 °C/min. The glass transition temperature (*T*_g_) was determined from the midpoint of the transition observed in the second heating cycle.

#### Modulated DSC (MDSC)

The *T*_g_ of the trilayer membranes was determined by using modulated differential scanning calorimetry (MDSC) on a Mettler Toledo DSC 5+ 1STZARe instrument. For the dry-state measurement, samples were dried overnight in a vacuum oven at 60 °C and subsequently cooled down at room temperature in a desiccator under vacuum. Conditioned samples were prepared by exposing only the TP side (or the SBS side) of the trilayer membranes to RH ∼98% for 1 week before testing. All experiments were performed under a nitrogen flow of 60 mL min^–1^. The temperature was increased from −80 to 120 °C at a heating rate of 2 °C min^–1^, using a modulation amplitude of ± 0.2 °C and a period of 1 min. The *T*_g_ was determined from the midpoint of the transition observed in the reversing signal of the heating flow.

#### Water Uptake

The water uptake of the various films and membranes was determined at room temperature (23 °C). The samples were cut into 1 × 1 cm^2^ pieces and dried in an oven at 60 °C overnight. The dry weight (*M*_d_) was measured before the samples were immersed in deionized (DI) water. The weight of the samples was determined in intervals until the point of equilibrium swelling had been reached (typically after 96 h); the weight of the fully swelled sample (*M*_∞_) was then recorded, and the water uptake was calculated using

1 $$water\;uptake\;\left(\%\right)=\frac{M_{\infty }-M_{d}}{M_{d}}\times 100$$

All water uptake data reported are averages from triplicate measurements, along with their standard deviations.

#### Water Permeability

Pyne permeability cups, manufactured by BEVS Industrial, were utilized to determine the water vapor permeability of the various membranes using standard ASTM E96. The cups were made from aluminum and had a test area (A) of 9.7 cm^2^. The membranes were cut into circles with a diameter of 6 cm. Before the membranes were mounted in the permeability cup, they were preconditioned under test conditions for a minimum of 2 days. The cup was loaded with either CaCl_2_ (dry-cup method) or DI water (wet-cup method), as shown in Figure S5. For the dry-cup method, the relative humidity on the donor side (RH_D_) was set to a steady value of RH_D_ ∼ 75% by placing a saturated solution of sodium chloride into the incubator in which the experiment was conducted.^34^ For the wet-cup method, a large quantity of dry calcium chloride was introduced into the incubator to maintain the receiver compartment at a relative humidity of nominally RH_R_ = 0%. The cups were removed in regular time intervals (1 < Δ*t* < 72 h) and weighed. The water vapor transmission rate (WVTR) was then determined from the change in mass (*g*) as a function of time (*t*), divided by the transport area (*A*)­

2 $$\mathrm{WVTR}=\frac{g}{A\times t}$$

At least five data points over a period of at least 24 h were analyzed by linear regression. The water vapor permeability (WP) was then determined by

3 $$\mathrm{WP}=\frac{\mathrm{WVTR}\times l}{\Delta p}$$

where *l* and Δ*p* are the thickness and water vapor pressure difference between the two sides of the membrane, respectively. The value of Δ*p* can be derived from the difference in relative humidity between the donor (RH_D_) and the receiver (RH_R_) compartments

4 $$\Delta p=\left({RH}_{D}-{RH}_{R}\right)p_{sat}$$

where *p*_sat_ is the saturated water vapor pressure at *T* = 25 °C (*p*_sat_ = 3162 Pa).

The water vapor permeability was measured in two directions, i.e, each side of the respective membrane once facing the humid donor side. The reported values are the mean and standard deviation of experiments carried out with *n* = 3 membranes for each composition. The asymmetry of the water transport was expressed by the asymmetry factor (AF)

5 $$AF=\frac{{\mathrm{WP}}_{SBS-TPx\to SBS}}{{\mathrm{WP}}_{SBS\to SBS-TPx}}$$

where WP_SBS‑TPx→SBS_ and WP_SBS→SBS‑TPx_ are the water permeabilities of bilayer and trilayer membranes measured in the direction from the SBS-TP or the TP side to the SBS side and vice versa, respectively.

## Results and Discussion

The membranes investigated in this study were laminates of films made from the hydrophobic, elastic polymer SBS, a previously reported hydrophilic copolymer made from HEMA, HEA, and EHMA (TP, Figure S1),^32^ or blends of SBS and TP, referred to as SBS-TP_*x*_. The weight fraction of the terpolymer, indicated by the subscript “x” was varied between 10 and 41% w/w. A higher TP content led to phase separation and the poor mechanical integrity of the resulting membranes. The terpolymer itself was synthesized as previously reported, with HEMA, HEA, and EHMA fractions of 55, 28, and 17 mol %, respectively (Figure S2), a number-average molecular weight (*M*_n_) of 56,000 g/mol (Figure S3), and a glass transition temperature (*T*_g_) of 62 °C (determined by differential scanning calorimetry, Figure S4). Films of neat SBS and neat TP, as well as various SBS-TP_*x*_ blends, were produced by solvent casting from THF and subsequent compression molding. The latter process was applied to produce smooth membranes with a homogeneous thickness of about 150 μm (Table 1). The pictures in Figure 1 demonstrate that these films are rather transparent. Their morphology was investigated based on scanning electron microscopy (SEM) (Figure 2) and optical microscopy images (Figure S6). The SEM images of cross sections of neat SBS (Figure 2a) and TP films (Figure 2f) reveal smooth, homogeneous morphologies, while the corresponding images of SBS-TP_*x*_ blend films (Figure 2b–e) reflect rough fracture surfaces that are characteristic of heterogeneous, phase-separated blends. Optical microscopy images, however, do not show any prominent features that reveal different phases, suggesting that the domain sizes are small (Figure S6).

**Figure 1:**
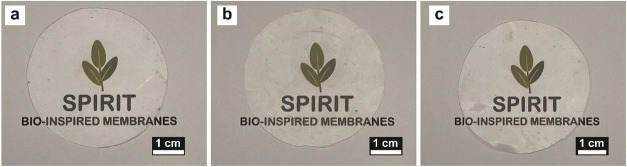
Digital photographs of (a) a neat SBS film, (b) a blend film of SBS and 41 wt % of the terpolymer poly­(HEMA-*co*-HEA-*co*-EHMA) (SBS-TP_41_), and (c) a neat TP film.

**Figure 2:**
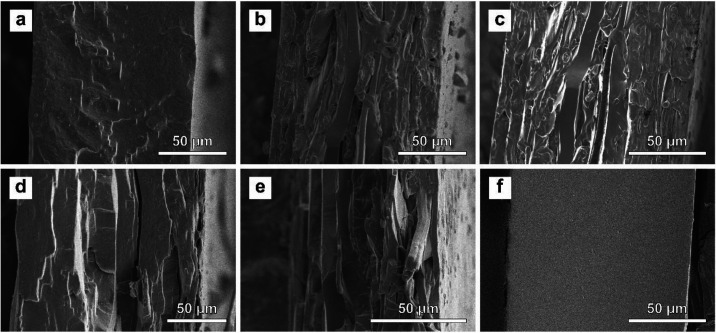
Scanning electron microscopy (SEM) images of the cross section of cryo-fractured films of (a) neat SBS, (b) SBS-TP_10_, (c) SBS-TP_23_, (d) SBS-TP_33_, (e) SBS-TP_41_, and (f) neat TP.

The mechanical properties of all films were investigated by dynamic mechanical analyses (DMA, Figure 3a) and tensile tests (Figure 3b); the key data are compiled in Table 2. The DMA trace of the neat SBS shows the expected features, notably the glass transitions of the poly­(butadiene) (PB) and poly­(styrene) (PS) domains at *T*_g_ **≈** −83 and 97 °C (maxima of the tan δ trace), respectively. The storage modulus (*E′*) of SBS assumes values of ca. 2.8 GPa in the glassy regime (−100 °C) and ca. 57 MPa in the rubbery plateau (20 °C). The DMA trace of the neat TP shows an extended glassy regime, with an *E′* of ca. 2.1 GPa (20 °C) and a *T*_g_ of 83 °C; note that this value is ca. 20 °C higher than the one established by DSC (Figure S4). The DMA traces of the SBS-TP_*x*_ blends are combinations of those of the individual components, reflecting three glass transitions associated with PB, TP, and PS domains, and glassy and rubbery domains with *E*′ values that fall between those of the two neat polymers. While the *T*_g_ of the TP phase is reduced relative to that of the neat TP, especially in the blends with a low TP content, the DMA traces clearly support the conclusion that the blends are phase-separated.

**Figure 3:**
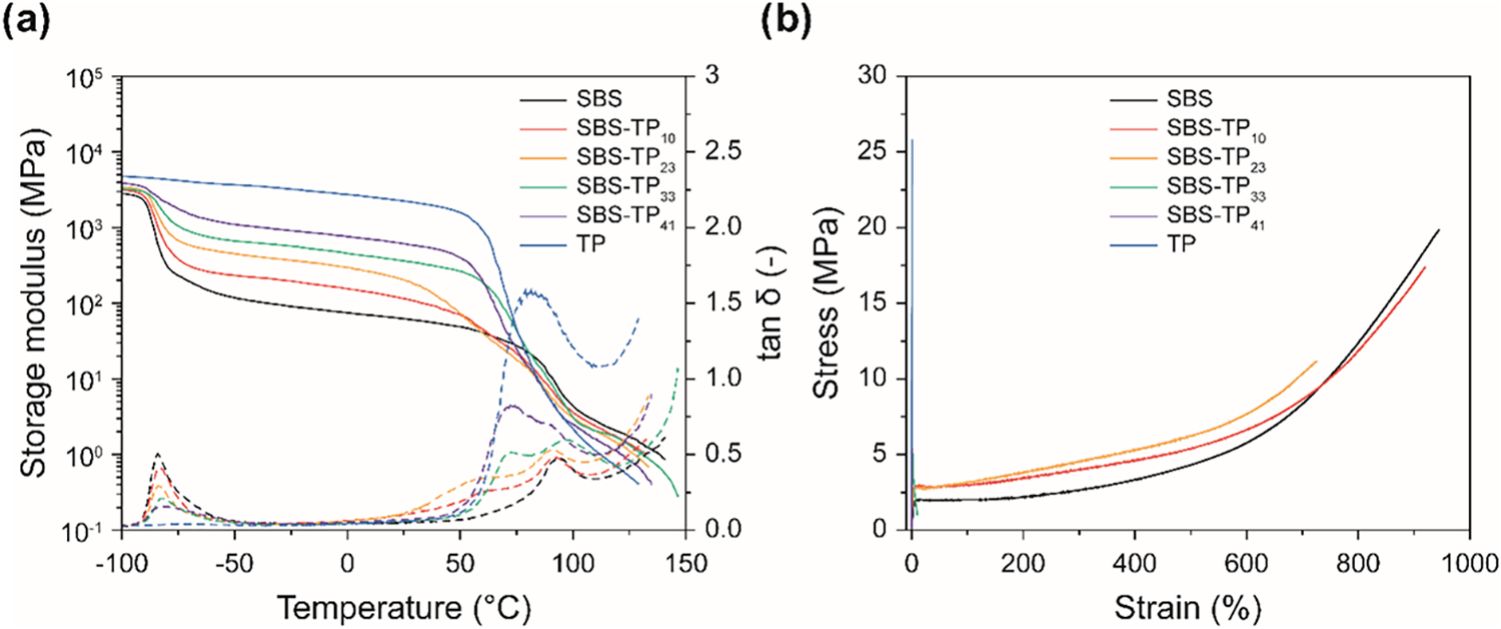
(a) Dynamic mechanical analysis (DMA) traces and (b) stress–strain curves of films of the neat SBS, the neat TP, and the SBS-TP_*x*_ blends. Tensile tests were conducted at room temperature at a strain rate of 50 mm/min.

**Table 2:** Mechanical Properties of Films of Neat SBS, Neat TP, and SBS-TP_*x*_ Blends

	glass transition temperature *T*_g_ ^a^,^b^ [°C]			^a^ ^c^	^d^		
sample	PB	TP	PS	storage modulus *E′* ^a^,^c^ [MPa]	Young’s modulus^d^ [MPa]	elongation at break ε_B_ ^d^ [%]	maximum strength σ_M_ ^d^ [MPa]
Neat SBS	–83		97	57 ± 13	54 ± 5	960 ± 15	20 ± 4
SBS-TP_10_	–83	50	95	121 ± 4	116 ± 7	927 ± 59	16 ± 1
SBS-TP_23_	–83	51	94	217 ± 26	164 ± 23	774 ± 42	11 ± 1
SBS-TP_33_	–82	74	96	433 ± 45	325 ± 47	16 ± 6	4.8 ± 0.8
SBS-TP_41_	–82	74	94	723 ± 88	495 ± 60	1.3 ± 0.6	5.3 ± 1.3
Neat TP		83		2488 ± 143	2140 ± 131	26 ± 3	1.3 ± 0.1

a Determined by dynamic mechanical analysis. All data represent averages of *n* = 3 individual samples ± standard deviation.

b The glass transition temperatures (*T*_g_) were determined from the maximum of the tan δ curves.

c At 20 °C.

d Determined by tensile tests at 23 °C. All data represent averages of *n* = 3 individual samples ± standard deviation.

The stress–strain curves of the neat SBS and TP films reflect the expected rubbery and glassy behaviors, respectively, with Young’s moduli *E* of 54 MPa and 2.1 GPa, maximum tensile strength σ_M_ of 20 ± 4 and 26 ± 3 MPa, and elongation at break *ε*_B_ of 960 ± 15 and 1.3 ± 0.1%, respectively (Figures 3b, S7). As expected, the introduction of TP into the SBS causes stiffening and embrittlement. Rather interestingly, however, blends SBS-TP_10_ and SBS-TP_23_ remain highly extensible with *ε*_B_ > 700%. A fundamental change is observed for blends with a TP content of 33 or 41 wt %, where *ε*_B_ drops to 16 and 1.3%, respectively, indicating that at high TP concentration, the rigid TP dominates the mechanical behavior.

The equilibrium water uptake of the various films is reported in Figure 4a. While the neat SBS hardly swells (0.0 ± 0.3% w/w), the TP shows a water uptake of 27.3 ± 0.3% w/w. The SBS-TP_*x*_ blends show a water uptake that increases quite linearly with the TP content. The dramatic difference in the mechanical characteristics between SBS-TP_23_ and SBS-TP_33_ is not reflected in the water uptake data, suggesting that equilibrium swelling is primarily driven by the TP fraction. The water vapor permeability (WP) of the various films was investigated as a function of relative humidity of the donor side (RH_D_) using dry cup (for RH_D_ = 75%) and wet cup (for RH_D_ = 100%) methods, while the relative humidity at the receiver side was kept at RH_R_ = 0% (Figure 4b). As previously reported,^24,27^ the WP of neat SBS films is low and independent of RH_D_, with an average value of 3.1 ± 0.2 × 10^–14^ kg m m^–2^ s^–1^ Pa^–1^. By contrast, the WP of the neat TP films is strongly humidity-dependent and increases by an order of magnitude from 4.6 ± 0.4 × 10^–14^ kg m m^–2^ s^–1^ Pa^–1^ at RH_D_ = 75% to 5.0 ± 0.9 × 10^–13^ kg m m^–2^ s^–1^ Pa^–1^ at RH_D_ = 100%, on account of the plasticization that the aqueous swelling imparts. The WP of the SBS-TP_*x*_ blend films shows the same behavior, i.e., in all cases, the WP increases considerably when RH_D_ is increased from 75 to 100%.

**Figure 4:**
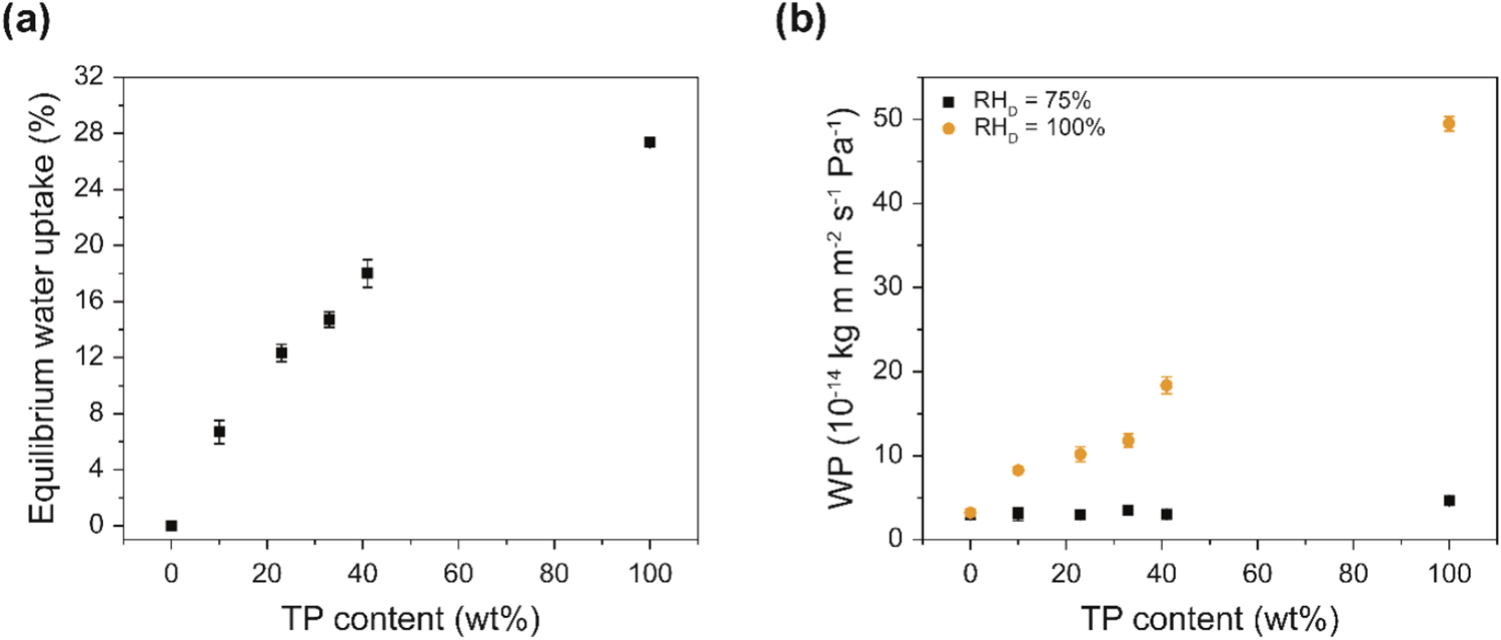
(a) Water uptake and (b) water permeability (WP) of the neat SBS, the neat TP, and the SBS-TP_*x*_ blend films. The water permeability measurements were carried out with a relative humidity at the donor side (RH_D_) of 75% or 100% and a relative humidity at the receiver side (RH_R_) of 0% using the dry-cup (75% RH_D_) and wet-cup (100% RH_D_) methods.

After confirmation that neat TP and SBS-TPx blend films show, as expected, a moisture-dependent WP, bilayer membranes composed of a neat SBS layer and a layer consisting of the neat TP or one of the SBS-TP_*x*_ blends were prepared by compression molding. Thus, individually prepared films with a thickness of ca. 150 μm were joined at 130 °C in a hot press fitted with a 300 μm thick spacer. Considering the moderate contrast in WP observed for the SBS-TP_10_ blend (Figure 4b), this composition was not used for the preparation of the bilayer membranes. The resulting bilayer membranes are all transparent, and no delamination was visible during handling. SEM images of the cross sections of Cryo-fractured membranes are shown in Figure 5. While the SEM images reveal delamination at the interface between SBS and the TP or the SBS-TP_*x*_ blends, this effect is less prominent as the TP content in the blend layer decreases, to the extent that delamination can no longer be discerned for the bilayer membrane made with SBS-TP_23_.

**Figure 5:**
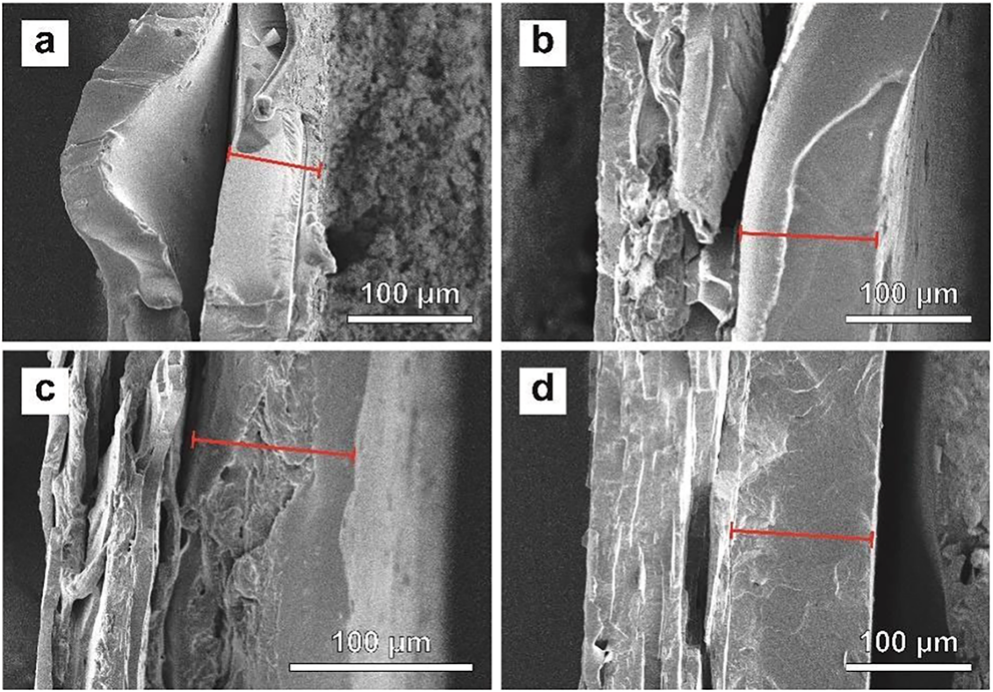
SEM image of the cross section of cryo-fractured bilayer membranes consisting of a layer of SBS and (a) the neat TP, (b) SBS-TP_41_, (c) SBS-TP_33_, and (d) SBS-TP_23_. In all SEM images, the SBS layer (red line) is positioned on the right side.

The mechanical properties of the bilayer membranes were investigated by tensile tests (Figure S8 and Table S2). The stress–strain curve of the SBS/TP bilayer membrane reflects the high stiffness and brittle failure of the entire membrane at a low *ε*_B_ (1.2 ± 0.4%) that appears to coincide with the fracture of the TP layer. By contrast, the SBS/SBS-TP_*x*_ membranes demonstrate an increased *ε*_B_, reflecting that the SBS layer continues to carry the load after the blend layer fails. While the SBS/SBS–TP_41_ and SBS/SBS–TP_33_ membranes fail at strains of 20 ± 11 and 72 ± 12%, respectively, the SBS/SBS–TP_23_ membranes are highly deformable, with an *ε*_B_ of 823 ± 54%. Overall, the mechanical data reflect an improved compatibility and mechanical integrity, as the neat TP is replaced with an SBS-TP_*x*_ blend.

The water uptake of the bilayer membranes increases with increasing TP content in the SBS-TP blend used to fabricate the membrane (Figure S9). As expected, the values are roughly half of those reported in Figure 4a for the corresponding blends or the neat TP. No delamination was observed upon immersion of either bilayer membrane in water, indicating reasonable adhesion between the TP (or the SBS-TP_*x*_ blend) and SBS layers. Considering that at RH_D_ = 75% the WP of the TP and the SBS-TP_*x*_ blends is not significantly higher than that of the neat SBS (vide supra), the WP of the various bilayer membranes was investigated only for RH_D_ = 100% and RH_R_ = 0%. To examine the potential directionality of the water transport, the WP of the bilayer membranes was measured as a function of the transport direction, i.e., with either the neat SBS or the TP (or SBS-TP_*x*_ blend) side facing the donor compartment (Figure 6a). Gratifyingly, all of the bilayer membranes investigated display asymmetric water transport characteristics. The WP is higher when the TP-containing side faces the donor and is plasticized and lower when the hydrophobic SBS layer faces the donor, thereby limiting the plasticization of the TP-containing layer. Figure 6b shows the AF values, i.e., the ratio of the water permeabilities measured along the two directions, determined for the various membranes. While the trend is subtle, the AF slightly increases with the TP content in the blend layer and reaches a maximum value of AF = 1.9 ± 0.1 for the SBS/TP bilayer membrane.

**Figure 6:**
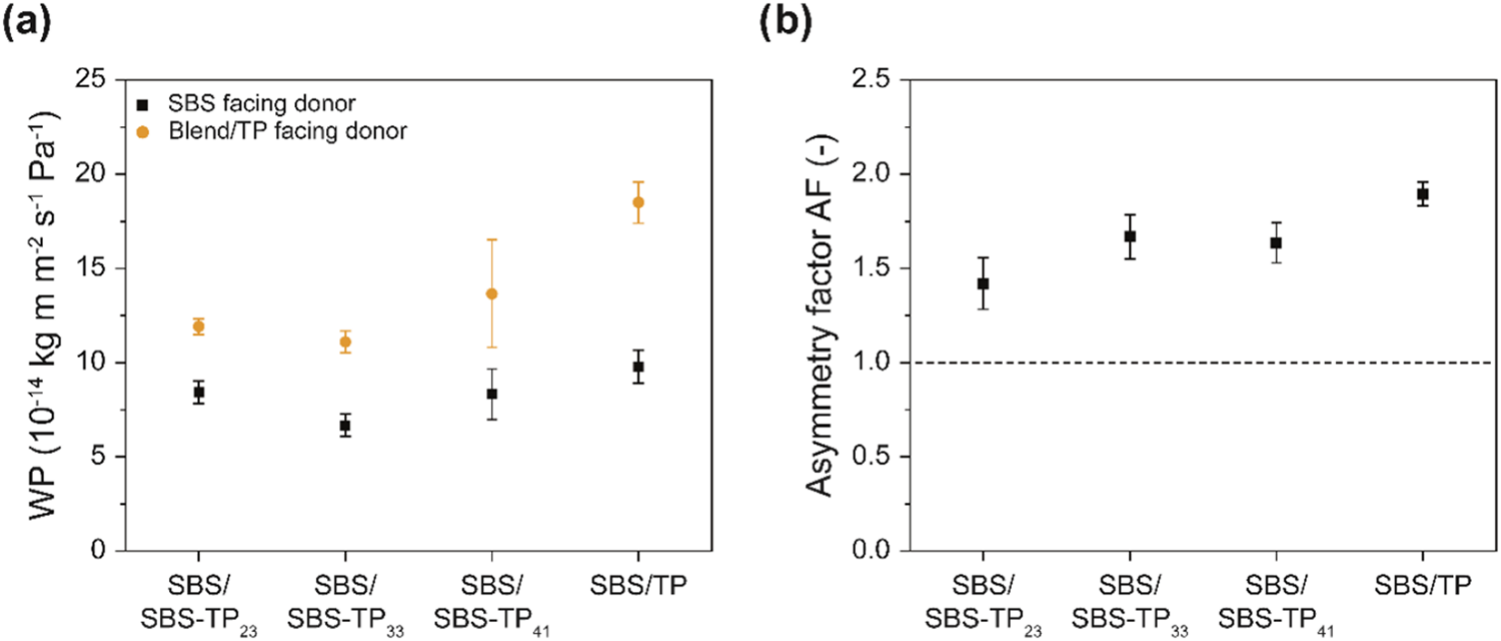
(a) Water permeability of SBS/TP and SBS/SBS-TP_*x*_ bilayer membranes as a function of direction, i.e., with either the SBS or the TP-containing layer facing the donor. Data were acquired with a relative humidity at the donor side (RH_D_) of 100% and a relative humidity at the receiver side (RH_R_) of 0%. (b) Asymmetry factor (AF) of the bilayer membranes.

Given the excellent adhesion between SBS-TP_23_ and SBS (Figure 5d), we sought to explore if the overall membrane performance could be further improved by creating SBS/SBS-TP_23_/TP trilayer membranes, in which a thin layer of the SBS-TP_23_ blend acts as an adhesion-promoting layer between neat SBS and TP layers. Thus, 50 μm thick SBS-TP_23_ films were sandwiched between 150 μm thick SBS and 150 μm thick TP films, and 300 μm thick trilayer membranes were fabricated by compression molding. SEM images of the cryo-fractured cross section of the membranes thus produced suggest intimate bonding between all three layers (Figure 7a). Tensile tests reveal that the dry SBS/SBS-TP_23_/TP membranes fracture at a lower strain than the SBS/TP bilayer membranes, confirming that the SBS-TP_23_ interlayer promotes efficient adhesion and stress transfer across the interfaces, resulting in a more cohesive deformation behavior (Figure S10, Table S3). On the other hand, the membrane becomes highly flexible after immersion in water, without any signs of delamination upon deformation, demonstrating its structural robustness under hydrated conditions (Supporting Movie S1). Thus, the intermediate SBS-TP_23_ layer prevents delamination observed in the bilayer membranes made from neat SBS and TP films (Figure 5a). To further support this, tensile tests were conducted on trilayer membranes conditioned at ∼98% relative humidity for 1 week (Figure S11). In comparison to the dry trilayer membrane, the moisture-conditioned specimens exhibit a reduction of approximately 1 order of magnitude in Young’s modulus (from 910 ± 66 MPa to 59 ± 12 MPa) and a pronounced increase in elongation at break (*ε*_B_ = 768 ± 53%). These results confirm that under hydrated conditions, the TP layer becomes plasticized, allowing the membrane to sustain substantially higher strain before failure (Figure S11).

**Figure 7:**
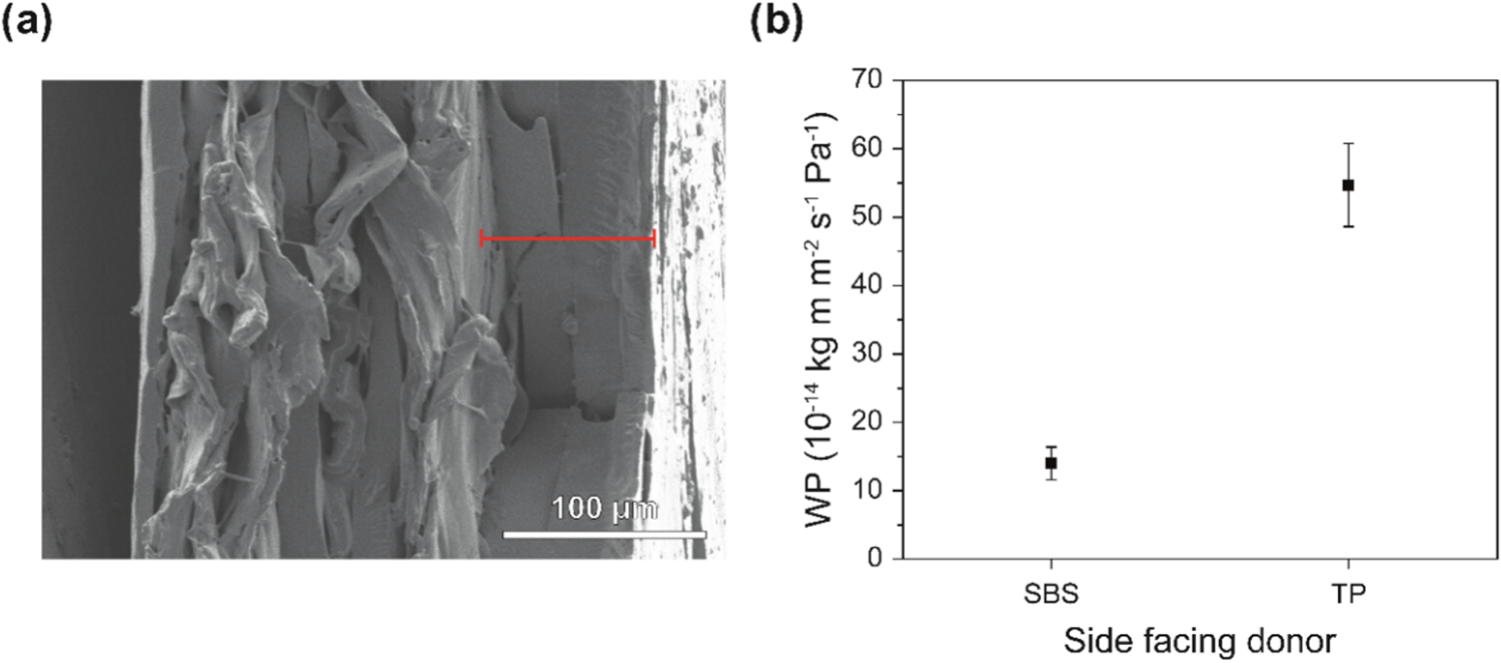
(a) Scanning electron microscopy (SEM) image of the cross section of a cryo-fractured SBS/SBS-TP_23_/TP trilayer membrane. The SBS layer (red line) is positioned on the right side. (b) Water permeability (WP) of an SBS/SBS-TP_23_/TP trilayer membrane as a function of direction, i.e., with either the neat SBS or the neat TP layer facing the donor. Data were acquired with a relative humidity at the donor side (RH_D_) of 100% and a relative humidity at the receiver side (RH_H_) of 0%.

The WP of the SBS/SBS-TP_23_/TP membranes was investigated for RH_D_ = 100% and RH_R_ = 0% as a function of the transport direction (Figure 7b). When the TP side faces the donor, the membranes exhibit a WP of 55.6 ± 6.1 × 10^–14^ kg m m^–2^ s^–1^ Pa^–1^, while in the opposite orientation, a WP of 14.2 ± 2.4 × 10^–14^ kg m m^–2^ s^–1^ Pa^–1^ is observed, resulting in an AF of 4.0 ± 1.1. This difference in WP reflects the asymmetric plasticization of the trilayer membranes, depending on which side is exposed to high relative humidity. This effect is supported by modulated DSC measurements performed on membranes exposed to ∼98% RH from either the TP or SBS side (Figure S12). Thermal modulation allowed separation of the total heat flow into reversing and nonreversing components, distinguishing the broad endothermic signal from water evaporation (nonreversing) from the heat capacity change associated with the glass transition of the TP layer (reversing) (Figure S12a). In the dry state, the *T*_g_ observed in the reversing flow matches that of the neat TP powder (*T*_g_ = 62 °C). Upon exposure to high RH, however, *T*_g_ shifts to lower temperatures, with the extent of the decrease depending on the exposed side (Figure S12b). When humidity is applied from the SBS side, *T*_g_ decreases to ca. 28 °C, whereas exposure from the TP side reduces it to below room temperature (*T*_g_ = −19 °C), indicating an enhanced chain mobility consistent with the higher WP measured when the TP side faces the donor compartment. The significant increase in the AF, compared to the SBS/TP bilayer membranes, may be related to a more uniform interface between the layers, which in turn leads to improved water transport through the membrane when the TP is hydrated, i.e., when the TP side faces the donor compartment. Note that the AF value of 4.0 ± 1.1 exceeds that of many of the previously investigated graded membranes (Table S4).^15^ In the context of directional moisture transport, the majority of AF values reported in the literature are below 4, with only a few notable exceptions. Specifically, the bilayer membrane composed of PVA and poly­(ethylene terephthalate) (PET) developed by Matsuno et al. in 1987 also exhibits an AF = 4.0,^20^ while only two systems, the graded poly­(ethylene-*graft*-vinyl alcohol) membranes reported by Rogers in 1965 ^26^ and the PVA-SBS bilayer membranes investigated by Grillo and Weder,^28^ exhibit slightly higher values. According to the survey reported in Table S4,^9,16,20,24,26–28,35–41^ the AF value of 4.0 ± 1.1 reported here ranks among the highest values documented for directional moisture-transport membranes to date.

## Conclusions

In summary, we investigated the structure–property relationships of asymmetric membranes based on the hydrophobic thermoplastic elastomer SBS and a hydrophilic terpolymer containing HEMA, HEA, and EHMA, as well as blends of SBS and the terpolymer. The SBS-TP blends exhibit no signs of macroscopic phase separation, and their mechanical and moisture-transport characteristics can be varied over wide ranges by simple compositional variation. All TP-containing films exhibit humidity-dependent transport characteristics, which render the moisture transport through bilayer membranes made from SBS and TP or the SBS-TP blends directional, with a maximum AF value of 1.9. Building upon these outcomes, we prepared trilayer membranes in which a thin SBS-TP_23_ layer was originally devised as an adhesion promoter. Interestingly, this approach not only mitigated delamination issues observed in bilayers and facilitated stress transfer across layers but also increased the AF to a value of 4.0, possibly due to a more uniform interface between the layers. We speculate that this strategy may be generalizable, not only to other trilayer systems but also to membranes with a larger number of layers, which would lead to a more gradual polarity change.

## Supplementary Material





## Data Availability

The raw data underlying the findings of this work can be found at 10.5281/zenodo.16993021.

## Notes

Experimental procedures, synthetic details, and analytic data, including ^1^H NMR spectra, SEC traces, additional mechanical and moisture-transport data, and tables with additional data (PDF)
Movie demonstrating the structural robustness of the SBS/SBS-TP23/TP membrane under hydrated conditions (MP4)
