# Supplementary material for: Directional Moisture Transport in Compositionally Graded Multilayer Membranes
Source: ACS Appl Polym Mater. 2025 Dec 8;7(24):16628–36. doi: 10.1021/acsapm.5c03299 (PMC12750524; doi:10.1021/acsapm.5c03299)
Supplement: Supplementary file 1 [file ap5c03299_si_001.pdf]

Supporting Information

Directional moisture transport in compositionally graded multilayer membranes

Natthakan Rattanaphong[a], Luca Grillo[b], Christoph Weder[a,b*], Stephan Thierry Dubas[a,c,d*]

[a]The Petroleum and Petrochemical College, Chulalongkorn University, Bangkok 10330, Thailand

[b]Adolphe Merkle Institute University of Fribourg, Chemin des Verdier 4, 1700 Fribourg, Switzerland

[c]Center of Excellence on Petrochemical and Materials Technology, Bangkok 10330, Thailand

[d]Machine Learning for Polymers and Materials Discovery Research Unit, The Petroleum and Petrochemical College, Chulalongkorn University, Bangkok 10330, Thailand

AUTHOR INFORMATION

Corresponding Authors

Christoph Weder - *Adolphe Merkle Institute, University of Fribourg, Chemin des Verdiers 4, 1700 Fribourg, Switzerland;* orcid.org/0000-0001-7183-1790; Email: christoph.weder@unifr.ch

Stephan Dubas - *The Petroleum and Petrochemical College, Chulalongkorn University. Bangkok 10330, Thailand and Machine Learning for Polymers and Materials Discovery Research Unit, The Petroleum and Petrochemical College, Chulalongkorn University, Bangkok 10330, Thailand; orcid.org/0000-0002-7188-3096; Email: Stephan.d@chula.ac.th*

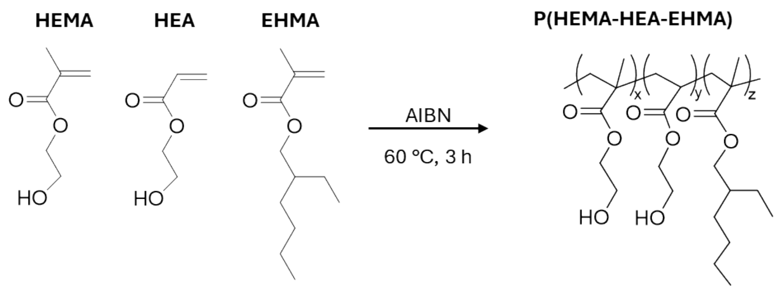

Figure S1. Schematic of the synthesis of poly(HEMA-*co*-HEA-*co*-EHMA) (TP).

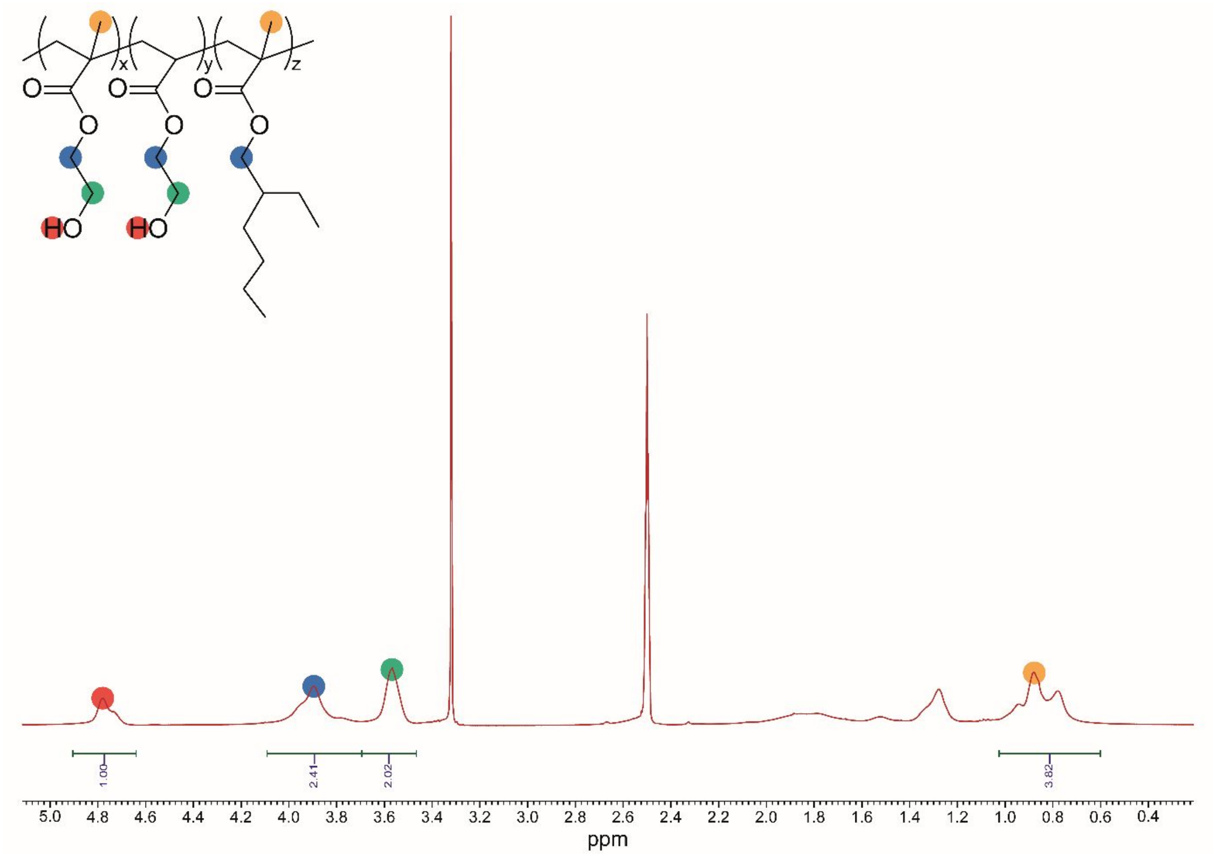

Figure S2. ^{1}H NMR spectrum of the (2-hydroxyethyl methacrylate-*co*-2-hydroxyethyl acrylate-*co*-2-ethylhexyl methacrylate) terpolymer (TP) in DMSO-d6.

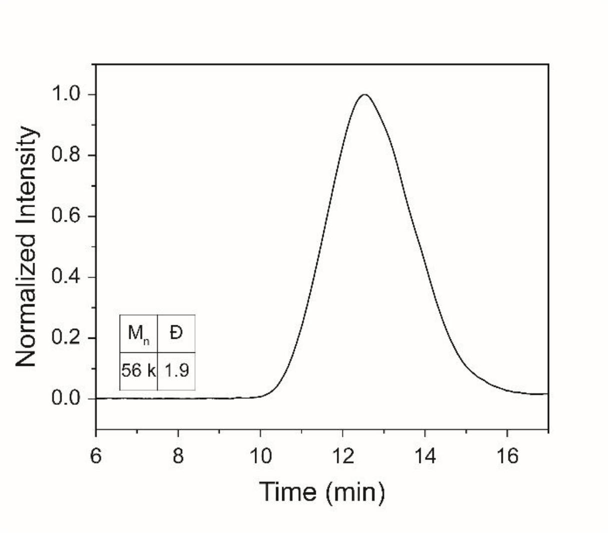

Figure S3. SEC chromatogram of the (2-hydroxyethyl methacrylate-*co*-2-hydroxyethyl acrylate-*co*-2-ethylhexyl methacrylate) terpolymer (TP) showing the normalized intensity as a function of elution time. The analysis afforded a number-average molecular weight (M_n) of 56,000 g/mol and a dispersity (Đ) of 1.9**.**

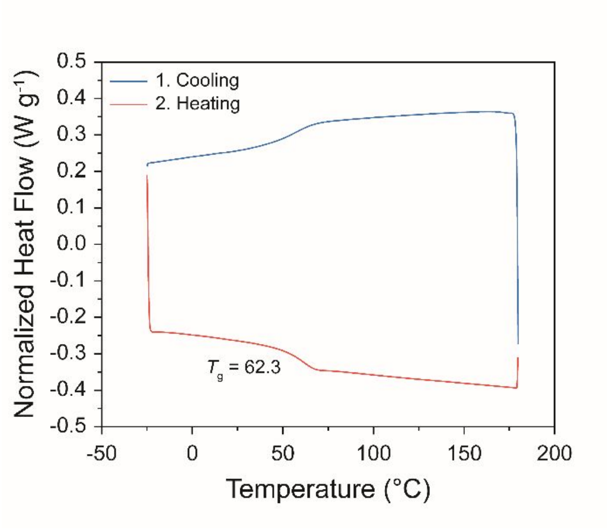

Figure S4. Differential scanning calorimetry (DSC) thermogram of the (2-hydroxyethyl methacrylate-*co*-2-hydroxyethyl acrylate-*co*-2-ethylhexyl methacrylate) terpolymer (TP) showing the first cooling and second heating cycles. The glass transition temperature (T_g) was determined to be 62 °C from the midpoint of the transition observed during the second heating cycle.

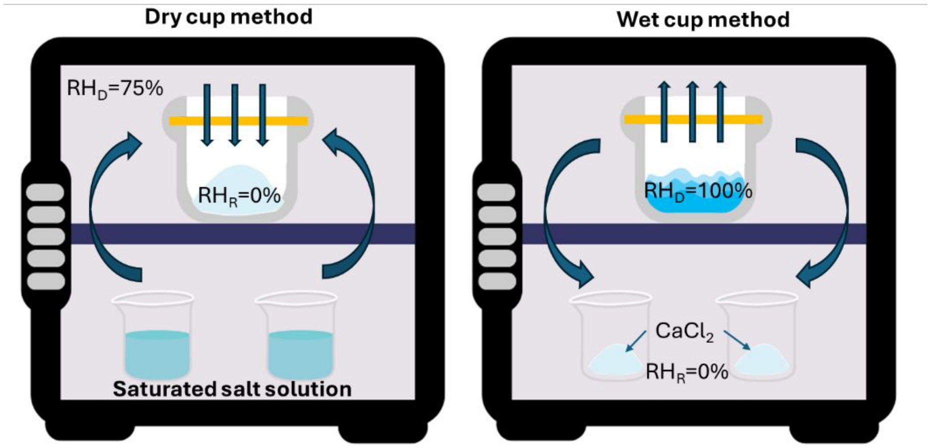

Figure S5. Schematic representations of the dry cup and wet cup methods used to measure the water permeability of the various membranes with a relative humidity at the donor side (RH_D) of 75 and 100%, respectively, and a relative humidity at the receiver side (RH_R) of ca. 0%.

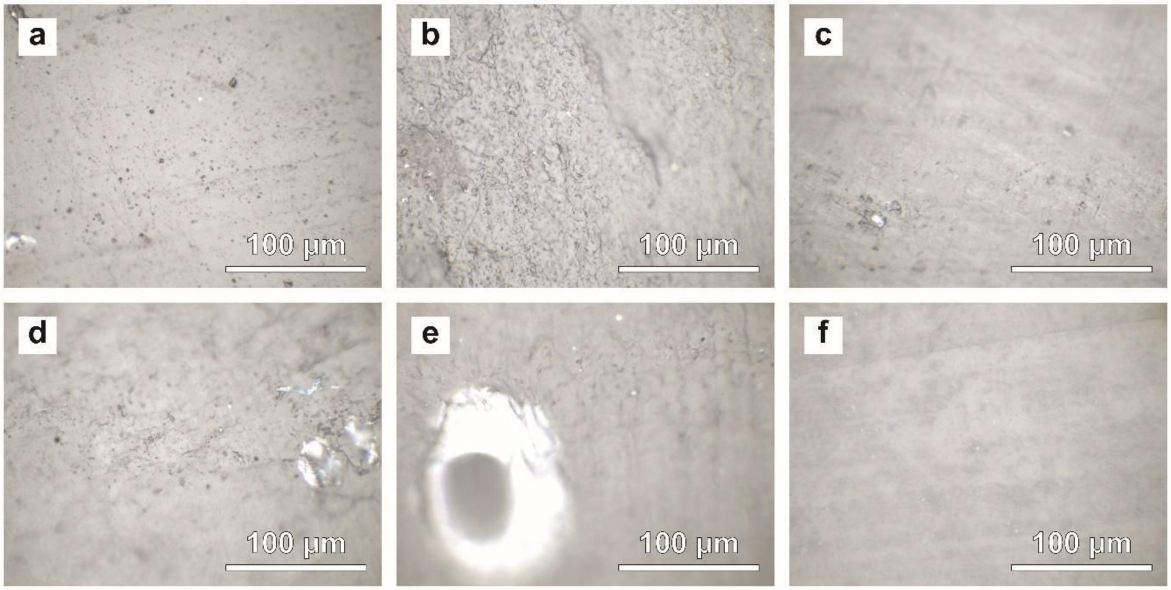

Figure S6. Optical microscopy images of (a) the neat SBS, (f) the neat TP, and the SBS-TP_x blends: b) SBS-TP_{10}, c) SBS-TP_{23}, d) SBS-TP_{33}, and e) SBS-TP_{41}.

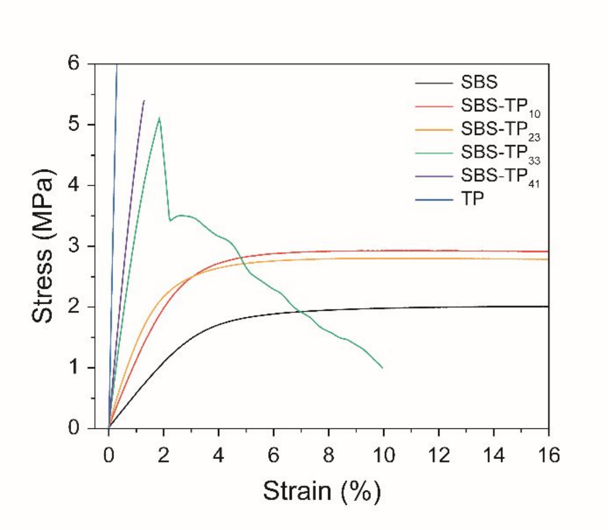

Figure S7. Magnification of the low-strain region of the stress-strain curves of films of the neat SBS, the neat TP, and SBS-TP_x blends. Tensile tests were carried out at room temperature at a strain rate of 50 mm/min.

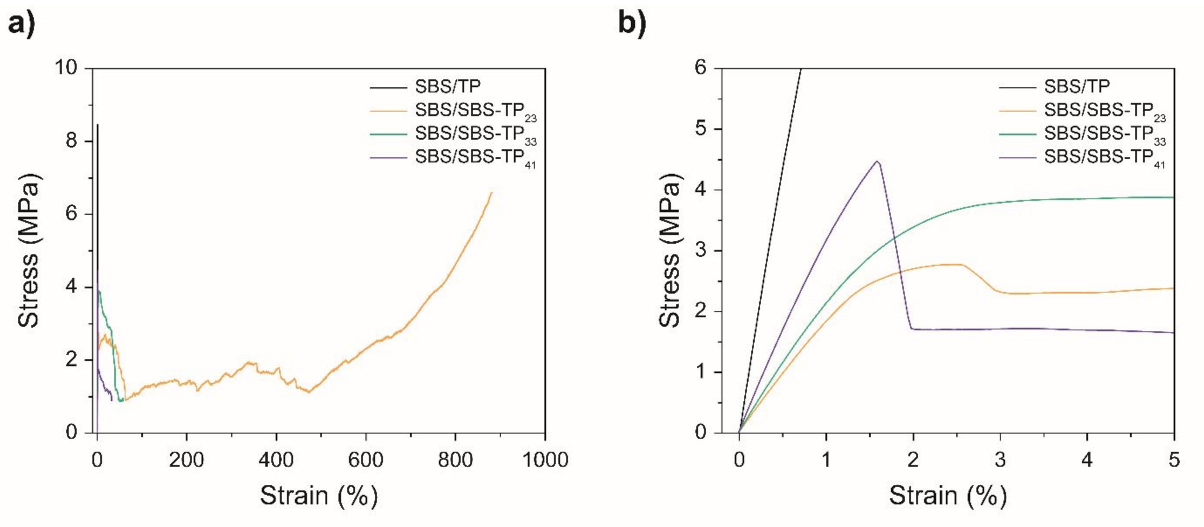

Figure S8. Stress–strain curves of SBS/TP and SBS/SBS-TP_x bilayer membranes. a) Entire stress-strain curves. b) Magnification of the low-strain region.

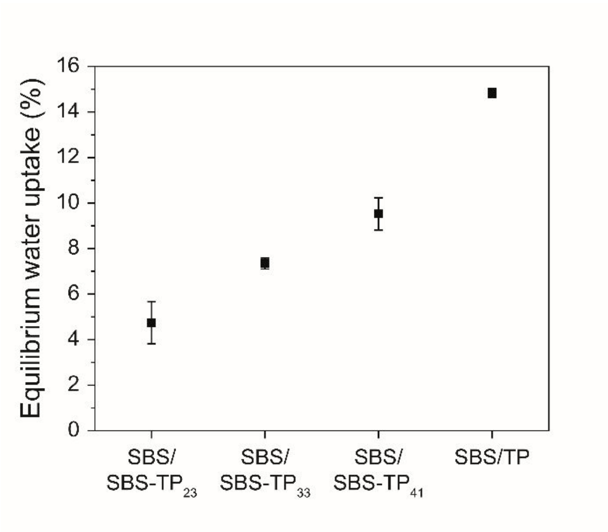

Figure S9. Water uptake of SBS/TP and SBS/SBS-TP_x bilayer membranes.

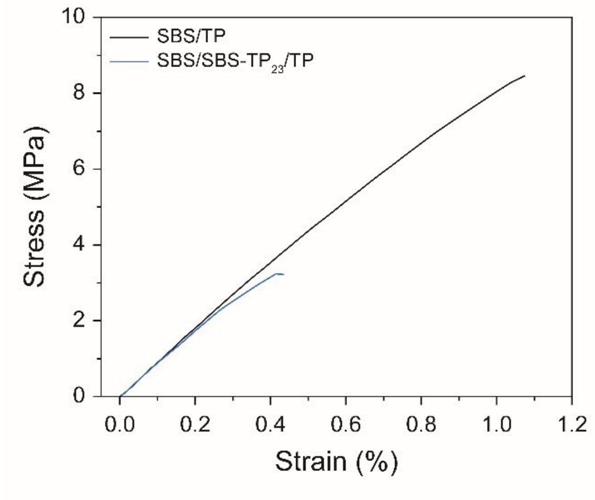

Figure S10. Stress-strain curves of the SBS/TP bilayer and the SBS/SBS-TP_{23}/TP trilayer membrane.

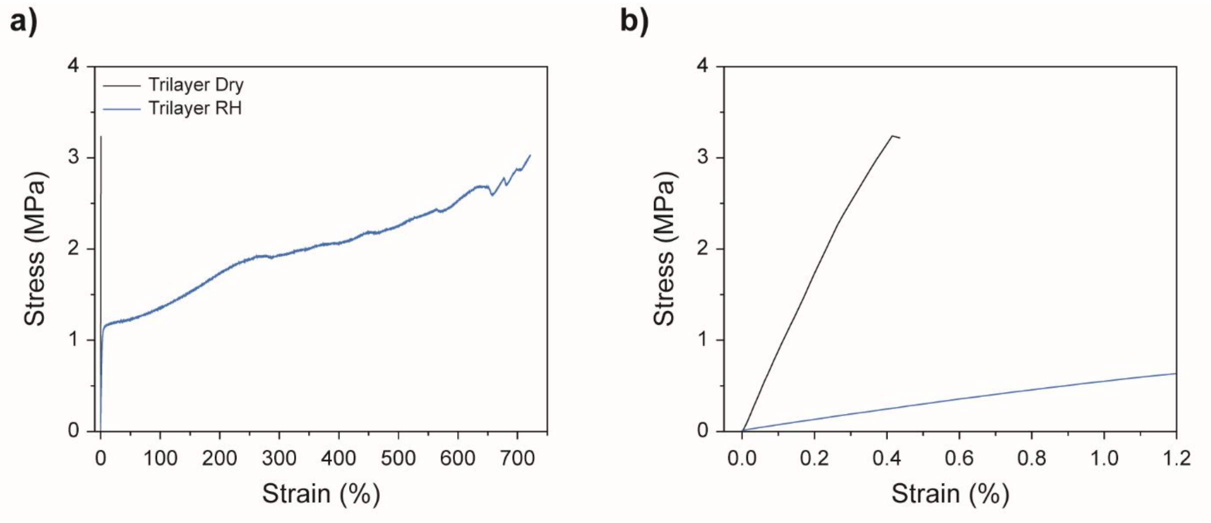

Figure S11. Stress-strain curves of the SBS/SBS-TP_{23}/TP trilayer membrane under dry conditions and after conditioning at RH ~ 98% for one week. a) Entire stress-strain curves. b) Magnification of the low-strain region.

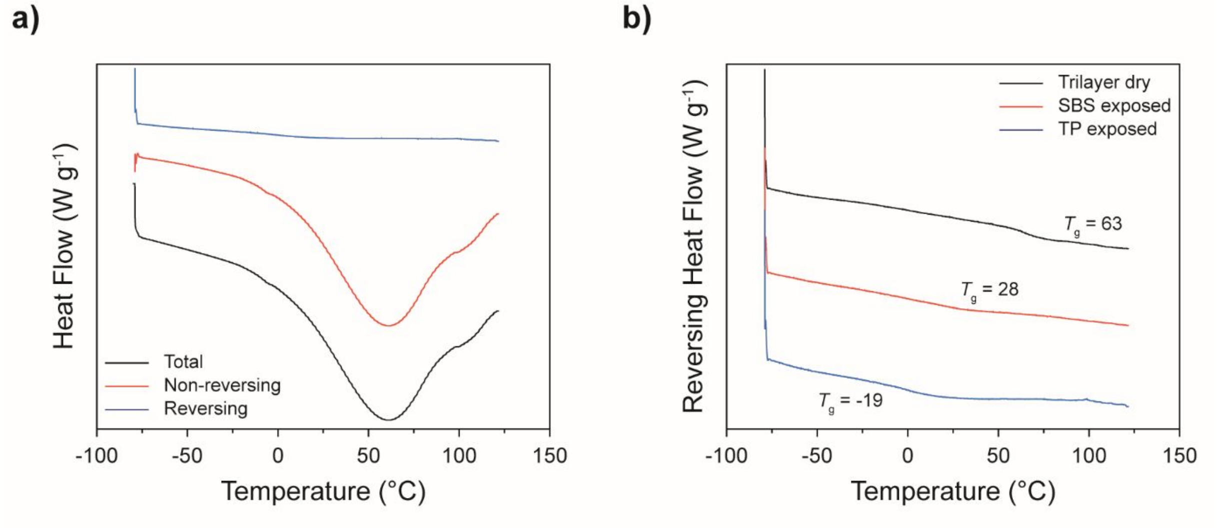

Figure S12. a) Modulated differential scanning calorimetry (MDSC) thermograph of a SBS/SBS-TP_{23}/TP trilayer membrane with the TP side exposed to RH ~ 98% for one week. The total (black), reversing (blue), and non-reversing (red) heat flow signals are shown. b) Reversing heat flow traces acquired by MDSC of trilayer membranes in the dry state (black) and with either the SBS (red) or TP (blue) side exposed to equilibrated RH ~ 98% for one week. During the MDSC experiment, the sample was heated from –80 °C to 120 °C at a rate of 2 °C min^{-1} under a nitrogen atmosphere, using a temperature modulation amplitude of ±0.2 °C and a modulation period of 60 s.

Table S1. Thickness of SBS and SBS-TP blend before and after compression as a bilayer membrane

Sample	Thickness of the neat SBS layer	Thickness of SBS-TP blend layer	Thickness of SBS/SBS-TP blend bilayer
**SBS/SBS-TP$_{23}$**	**162±9**	**157±12**	**258±11**
**SBS/SBS-TP$_{33}$**	**169±9**	**162±14**	**269±7**
**SBS/SBS-TP$_{41}$**	**166±7**	**166±12**	**302±13**
**SBS/TP**	**171±10**	**152±9**	**318±15**

Table S2. Mechanical properties of SBS/SBS-TP blend bilayers

Sample	Young's modulus[a] [MPa]	Elongation at break ε_B [a] [%]	Maximum stress σ_M[a] [MPa]
**SBS/SBS-TP$_{23}$**	194±32	823±54	5±1
**SBS/SBS-TP$_{33}$**	227±34	72±12	4±1
**SBS/SBS-TP$_{41}$**	346±12	20±11	4±1

[a]Determined by tensile tests at 23 °C. All data represent averages of n=3 individual samples ± standard deviation.

Table S3. Mechanical properties of the SBS-TP bilayer and the SBS/SBS-TP$_{23}$/TP trilayer membrane.

Sample	Young's modulus[a] [MPa]	Elongation at break ε_B [a] [%]	Maximum stress σ_M[a] [MPa]
**SBS/TP**	974 ± 86	1.2 ± 0.4	11 ± 3
**SBS/SBS-TP$_{23}$/TP**	910 ± 66	0.5 ± 0.2	3.6 ± 0.4

[a]Determined by tensile tests at 23 °C. All data represent averages of n=3 individual samples ± standard deviation.

Table S4. Overview of artificial dense membranes exhibiting directional moisture transport properties with the respective fabrication method and maximum asymmetry factor (AF) obtained.

Year	Membrane	Fabrication	Max Asymmetry factor (*AF*)	Ref.
1957	Bilayer of polyamide 6 (Nylon 6) and ethyl cellulose (Ethocell 610)	Combination of free-standing membranes in series	3.4	16
1965	Graded poly(ethylene) (PE) membranes grafted with vinyl alcohol (PVA)	One-sided diffusion of vinyl acetate (VAc) in PE and radiation-induced polymerization. Hydrolysis of VAc to PVA	6.5	26
1968	Graded poly(ethylene) (PE) membranes grafted with 2-vinylpyridine (2VP)	Radiation-induced 2VP grafting on PE followed by one-sided quaternization with methyl bromide vapors	2.0	35
1971	Graded poly(styrene-*alt*-4-vinyl pyridine) membranes quaternized on a gradient	Quaternization of precast membranes with methyl bromide vapors	1.6	36
1978	Graded oxidized poly(L-methionine) (PLM) membranes	One-sided hydrogen peroxide treatment of PLM membranes	1.5	37
1983	Bilayer membranes of Neoprene (CR) and ethylene-propylene-diene terpolymer (EPDM)	Molding and lamination	1.2	38
1986	Bilayer membranes of styrene-butadiene rubber (SBR) and EPDM	Molding and lamination	1.8	39
1986	Bilayer membranes of EPDM and nitrile butadiene rubber (NBR)	Molding and lamination	1.6	40

Year	Membrane	Fabrication	Max Asymmetry factor (*AF*)	Ref.
1987	Bilayer membranes of poly(vinyl alcohol) (PVA) / poly(vinyl acetate) (PVAc), (PVA/PVAc)	Lamination by pressing preformed films	1.6	20
1987	Bilayer membranes of poly(vinyl alcohol)/ poly(ethylene terephthalate) (PET), (PVA/PET)	Lamination by casting of PVA on preformed PET film	4.0	20
2005	Pseudo-bilayered membranes of partially hydrolyzed poly(ethylene-*co*-vinyl acetate) (EVA)	One-sided alkaline hydrolysis treatment of EVA membranes	2.5	9
2021	Graded poly(styrene)-*block*-poly(butadiene)-*block*-poly(styrene) (SBS) / Cellulose nanocrystals (CNCs) (SBS/CNC) nanocomposite membranes	Solvent casting-evaporation and sedimentation of the colloidally unstable CNCs	3.0	24, 41
2021	Graded SBS/CNC and SBS/oleic acid-modified cellulose nanocrystals (OLA-CNCs)(SBS/OLA-CNC) nanocomposite membranes	Solvent casting-evaporation and sedimentation of the colloidally unstable OLA-CNCs	1.2	41
2024	Nanocomposite membranes with PVA nanofibers asymmetrically embedded in an SBS matrix	Solvent casting of SBS solution on a porous mat of electrospun PVA nanofibers	2.3	27
2025	Bilayer Membranes of PVA/SBS	Lamination *via* sequential solution casting of SBS solution on PVA film	5.8	28
